# T cell receptor sequences are the dominant factor contributing to the phenotype of CD8^+^ T cells with specificities against immunogenic viral antigens

**DOI:** 10.1016/j.celrep.2023.113279

**Published:** 2023-10-25

**Authors:** Daniel G. Chen, Jingyi Xie, Yapeng Su, James R. Heath

**Affiliations:** 1 Institute of Systems Biology, Seattle, WA 98109, USA; 2 Vaccine and Infectious Disease Division, Fred Hutchinson Cancer Research Center, Seattle, WA 98109, USA; 3 Clinical Research Division, Program in Immunology, Fred Hutchinson Cancer Research Center, Seattle, WA 98109, USA; 4 Molecular Engineering & Sciences Institute, University of Washington, Seattle, WA 98105, USA; 5 Department of Bioengineering, University of Washington, Seattle, WA 98105, USA; 6 These authors contributed equally; 7 Lead contact

## Abstract

Antigen-specific CD8^+^ T cells mediate pathogen clearance. T cell phenotype is influenced by T cell receptor (TCR) sequences and environmental signals. Quantitative comparisons of these factors in human disease, while challenging to obtain, can provide foundational insights into basic T cell biology. Here, we investigate the phenotype kinetics of 679 CD8^+^ T cell clonotypes, each with specificity against one of three immunogenic viral antigens. Data were collected from a longitudinal study of 68 COVID-19 patients with antigens from severe acute respiratory syndrome coronavirus 2 (SARS-CoV-2), cytomegalovirus (CMV), and influenza. Each antigen is associated with a different type of immune activation during COVID-19. We find TCR sequence to be by far the most important factor in shaping T cell phenotype and persistence for populations specific to any of these antigens. Our work demonstrates the important relationship between TCR sequence and T cell phenotype and persistence and helps explain why T cell phenotype often appears to be determined early in an infection.

## In brief

Chen et al. demonstrate that for immunogenic viral antigens, in both active infection and bystander activation contexts, TCR sequences are quantitatively the most important factor for antigen-specific CD8^+^ T cell phenotype and out-compete environmental signals such as plasma cytokines in terms of phenotypic impact.

## Graphical abstract

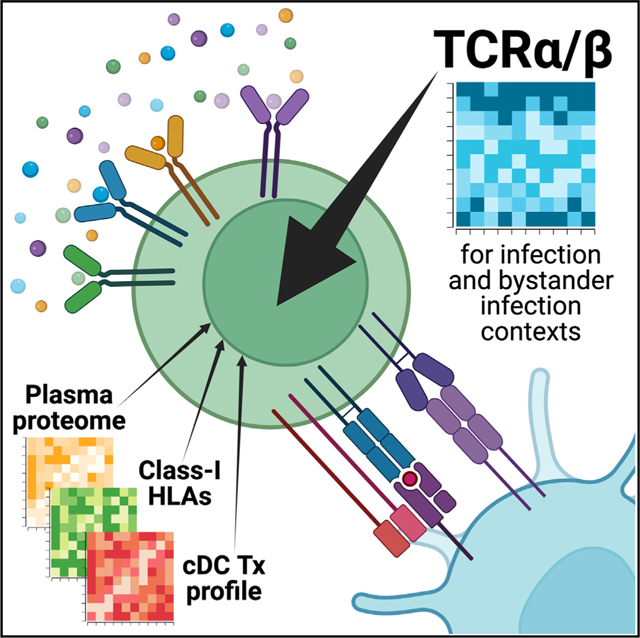

## INTRODUCTION

CD8^+^ T cells are one of the central classes of immune agents responsible for the clearance of pathogen-infected cells.^1^ Each T cell has a receptor (TCR) that largely dictates its specificity to peptide antigen-major histocompatibility complexes (pMHCs) and is known to influence phenotype.^2–4^ T cell phenotype may also be influenced by environmental factors, such as T cell-priming dendritic cells through co-receptor interactions or plasma proteins through cytokine signaling. Additionally, the HLA haplotype, which specifies the antigen-presenting MHCs, can also influence T cell phenotype through protein-protein interactions.^5–11^ While it is known that these factors can shape phenotype, their relative importance can be challenging to resolve, especially within the context of human disease.

Previous efforts to resolve this question have often employed model systems such as cell lines or mice and have typically focused on only one of the T cell-influencing factors. This not only prevents an analysis of the relative importance of these factors but also loses the context of human disease biology. Further, these past works have often not included antigen specificity of the T cells. Given that an immunogenic pMHC may promote the expansion of many different T cell clonotypes within a single patient or across patients, knowledge of antigen specificity may permit a direct measure of how variations in T cell α/β genes and other factors influence T cell phenotype.^3,12–15^

We interrogate the role of multiple factors, including TCRα/β genes, protein-protein interactions, and plasma proteome, that have been reported to influence the T cell clonotype-T cell phenotype relationship by studying 68 HLA-A*02:01 COVID-19 patients with full clinical and immune phenotyping. Within this cohort, we investigated clonotypes that were activated by an immunogenic severe acute respiratory syndrome coronavirus 2 (SARS-CoV-2) antigen, bystander-activated clonotypes specific to an immunogenic cytomegalovirus (CMV) antigen, and non-activated clonotypes specific to an immunogenic influenza antigen. In all cases, we found that the antigen-recognizing TCR sequences are the most influential factors in determining T cell phenotype. For the bystander-activated T cells, other factors play significant, albeit lesser, roles. Our work demonstrates that for immunogenic viral antigens, it is TCR sequences, not environmental signals, that primarily influence the phenotype and persistence of antigen-specific CD8^+^ T cell clonotypes both during and after infection.

## RESULTS

### Multi-omics analyses of antigen-specific CD8^+^ T cells for multiple epitopes in a longitudinal patient cohort

**Figure 1.**
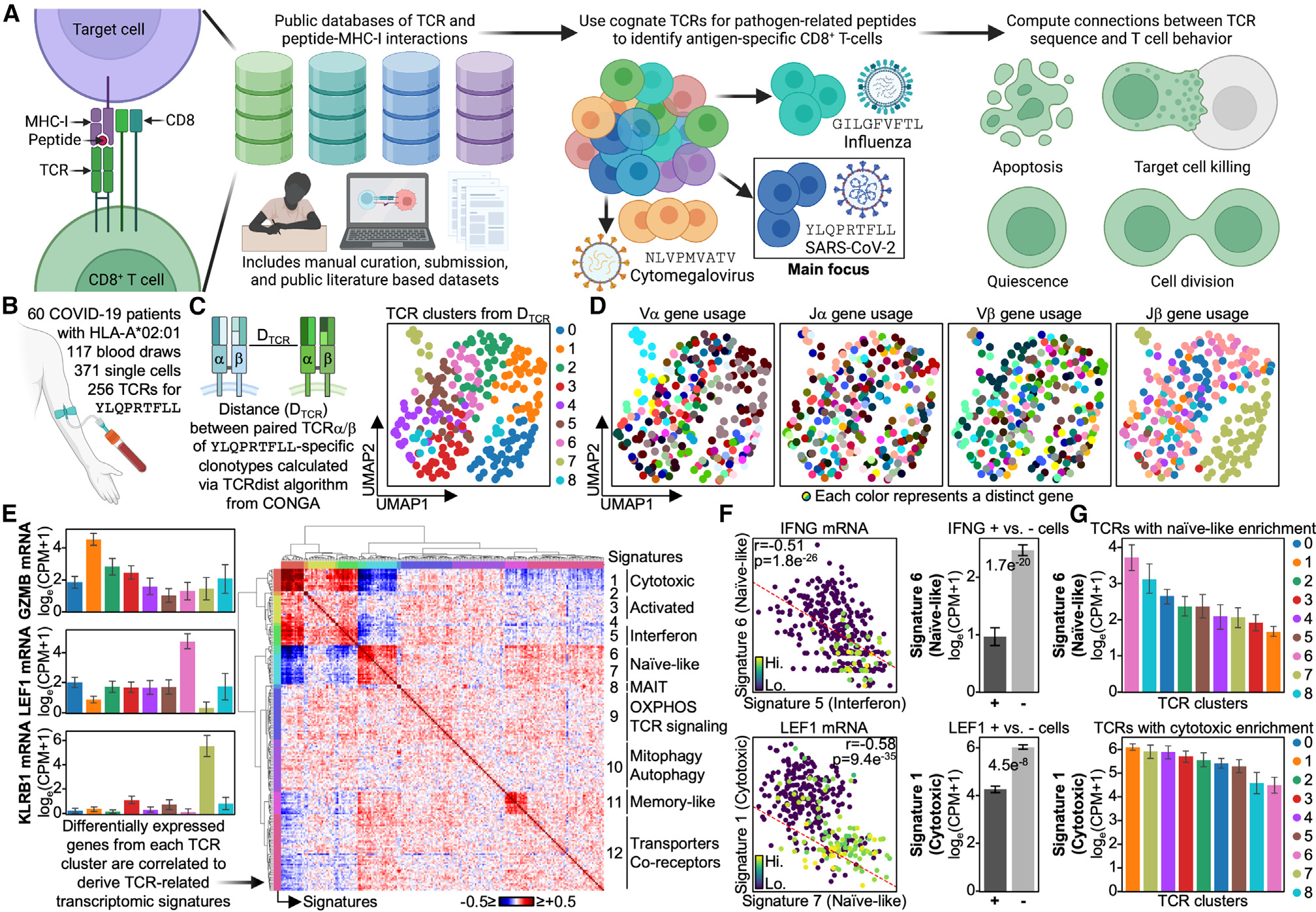
CD8^+^ T cells against the same antigen are diverse in phenotype and TCR (A) Public databases of pMHC-TCR interactions with CD8^+^ T cells are compiled to identify TCRs specific for YLQPRTFLL (SARS-CoV-2), NLVPMVATV (CMV), and GILGFVFTL (influenza) for HLA-A*02:01 donors. Public antigen-TCR pairs are utilized to identify antigen-specific CD8^+^ T cells, and their TCR sequence is correlated with their phenotypic behavior and transcriptome. (B) Longitudinal single-cell multi-omics dataset of HLA-A*02:01 COVID-19 patients is utilized to identify single YLQPRTFLL-specific CD8^+^ T cells via their TCRs. (C) Left: TCRs are clustered via their α- and β-chain amino acid sequences and TCR genes. Right: scatterplot of TCR-derived UMAP with TCR clusters colored (legend on the right). (D) Scatterplot of TCR-derived UMAP with variable (V) and junction (J) genes of α and β chains colored (legend on the bottom). (E) Left: bar plot of TCR clusters on the x axis and mRNA expression levels on the y axis. mRNAs are selected from differentially expressed genes (DEGs). Right: correlation matrix of the top 25 DEGs from each TCR cluster. Functional annotation based on enrichment and individual genes are on the right (legend on the bottom). (F) Left: scatterplot between single-cell mRNA signatures with select mRNAs plotted. Middle: bar plot of cells positive (+) and negative (−) for select mRNAs on the x axis and mRNA signature levels on the y axis. Right: bar plot of TCR clusters on the x axis and mRNA signature levels on the y axis. Significant p values are labeled on respective plots. Error bars on bar plots represent standard error, and the bar level represents the mean value of the given variable.

We analyzed antigen-specific CD8^+^ T cells from a longitudinal cohort of COVID-19 patients with varying acute disease severities as quantified through the WHO ordinal scale (Table S1.1).^16,17^ To measure patient responses across time, blood samples were taken at diagnosis (T1), several days after infection (T2), and during convalescence 2–3 months post-initial infection (T3). Antigen-specific CD8^+^ T cells were identified by using GLIPH2 to match TCRs from our dataset with pMHC-TCR pairs from public databases and literature (Figure 1A; Table S1.2; see STAR Methods).^18–21^ Starting from an initial patient cohort size of 209, we focused on 68 patients expressing the common HLA-A*02:01 allele^22,23^ because immunogenic antigens presented by this allele have been well characterized for multiple pathogens.^7,8,24–27^

We investigated CD8^+^ T cells exhibiting specificity to antigens derived from three viruses—SARS-CoV-2, CMV, and influenza. Each antigen has been reported as immunodominant for their respective virus.^7,8,24–27^ The first antigen, YLQPRTFLL (SARS2-YLQ), is derived from the spike protein of SARS-CoV-2, and T cells specific to this antigen are activated via antigen stimulation. The second antigen, NLVPMVATV (CMV-NLV), is derived from the pp65 protein of CMV. CMV-specific CD8^+^ T cells have been shown to be bystander activated (i.e., activated through soluble factor signaling rather than antigen stimulation) during COVID-19.^16,28^ The third antigen, GILGFVFTL (Flu-GIL), is from the M1 protein of influenza (Flu); CD8^+^ T cells specific to this antigen do not undergo reactivation during SARS-CoV-2 infection.^29^ Thus, T cells specific to these three immunogenic antigens represent three distinct populations during COVID-19 and so are expected to also exhibit unique phenotypic characteristics.

Throughout this article, we utilize the SARS2-YLQ-specific T cells for most of our analyses, as those cells are responding to the active SARS-CoV-2 infection and are thus most relevant to patient disease state. Where appropriate, parallel analysis of T cells specific to the CMV-NLV and Flu-GIL antigens was carried out to provide tests for the generality of our findings.

### CD8^+^ T cells against the same antigen are diverse in phenotype and TCR

We identified a total of 371 SARS2-YLQ-specific CD8^+^ T cells that consisted of 256 clonotypes from 117 longitudinal blood draws and 60 COVID-19 patients (Figure 1B). To compare antigen-specific TCR sequences with one another, we created a distance matrix using the well-accepted TCRdist algorithm to compare clonotypes by both TCR gene usage and amino acid sequence information from TCR α and β chains.^30^ We utilized this distance matrix to cluster clonotypes by TCR sequence similarity and created a two-dimensional projection of clonotypes via uniform manifold approximation and projection (UMAP) where similar TCR sequences are closer together (Figure 1C; Tables S1.3 and S1.4; see STAR Methods). Although these clonotypes target the same antigen, they form distinct clusters that are unique in TCR α- and β-chain gene usage, sequence, and GLIPH motifs (Figures 1D and S1A–S1C). For example, TCR-derived cluster 7 is distinct through its homogeneous use of TRAV1–2, a Vα gene commonly used by MAIT, mucosal-associated invariant T, cells.^31,32^ Unique TCR motifs for each cluster suggest that they differ in TCR structure, perhaps suggesting differences in downstream TCR signaling and thus differences in phenotype.

We interrogated for differences in phenotype between the TCR-derived clusters by performing differential gene expression analysis. For example, TCR cluster 1 was enriched for cytotoxic-associated *GZMB* expression, while cluster 6 had higher levels of the naive-associated *LEF1*^+^ (Figure 1E). This analysis further confirmed that cluster 7 was composed of MAIT cells through the unique upregulation of the MAIT-related transcript *KLRB1* (CD161).^32^

To enhance our understanding of the transcriptomic functions that separate these clusters, we correlated the top differentially expressed genes with one other to identify co-expressed gene modules (Figure 1E; Tables S1.5–S1.7; see STAR Methods). We did, in fact, observe clearly resolved cytotoxic, interferon, naive-like, and memory-like (Figure 1E) transcriptomic modules. As would be expected, the naive-like signature was anti-correlated with both interferon and cytotoxic signatures (Figure 1F). This verification added confidence to our use of these signatures to characterize the TCR-derived clusters. Further, the TCR-derived clusters exhibited statistically significant associations with these phenotypic signatures. The major axis of variation across the 12 TCR clusters was a naive-to-cytotoxic axis that highlighted TCR cluster 1 as the most cytotoxic and TCR clusters 0, 6, and 8 as the most undifferentiated (Figures 1G and S2A). Intriguingly, the MAIT cluster exhibited an upregulated cytotoxic signature (Figure 1G, bottom). Previous work has suggested that MAIT cells present during SARS-CoV-2 infection can become activated and are associated with disease severity.^33,34^ To confirm the effector-like phenotype of our MAIT cluster, we compared our TCR sequences with those from Shomuradova et al.,^25^ who identified SARS2-YLQ-specific interferon γ (IFNγ)-producing CD8^+^ T cells. In agreement with our findings, the TCR sequences of the IFNγ-producing clonotypes from literature could be mapped back to our MAIT cluster (Figure S2B).^25^ We do note that, while this literature pulled out MAIT-related sequences, in agreement with ours, via YLQ-specific pMHC tetramers, MAIT cells have been found to be non-selective binders and thus may not be binding SARS2-YLQ epitopes specifically. Thus, we suggest a hint of caution. Nonetheless, this agreement between our data and the literature demonstrates that our observations are, in part, verifiable through the literature. Overall, this analysis revealed a surprisingly broad phenotypic diversity, even though all of the clonotypes share the same target antigen.

We investigated whether the phenotypic diversity we observed for clonotypes targeting the same antigen was just a SARS2-YLQ-specific phenomenon or if was more general. In fact, a similar diversity was observed in CD8^+^ T cell populations specific to the Flu-GIL and CMV-NLV antigens, even though those T cells exhibit distinct phenotypic behaviors relative to the SARS2-YLQ-specific CD8^+^ T cells. When subjected to the same analytic pipeline performed on SARS2-YLQ-specific cells, both Flu-GIL- and CMV-NLV-specific CD8^+^ T cells presented with distinct TCR clusters that had divergent transcriptomic phenotypes with genes spanning multiple functional modules from IFN (e.g., *IFI44L*, *IFITM1*) to cytotoxic (e.g., *GNLY*, *PRF1*) to memory (e.g., *IL7R*) and naive (e.g., *TCF7*, *SELL*) (Figure S2C). We note, however, that the different antigen-specific populations did exhibit different biases toward these modules. For example, the CMV-specific T cells presented with more effector-related genes. Overall, these analyses show that CD8^+^ T cell clonotypes, even when specific for the same antigen, can exhibit significant variations in phenotype, and specific phenotype groupings can be associated with specific TCR sequence groupings.

### TCR sequence similarity correlates with phenotypic similarity for CD8^+^ T cell clonotypes against the same antigen

**Figure 2.**
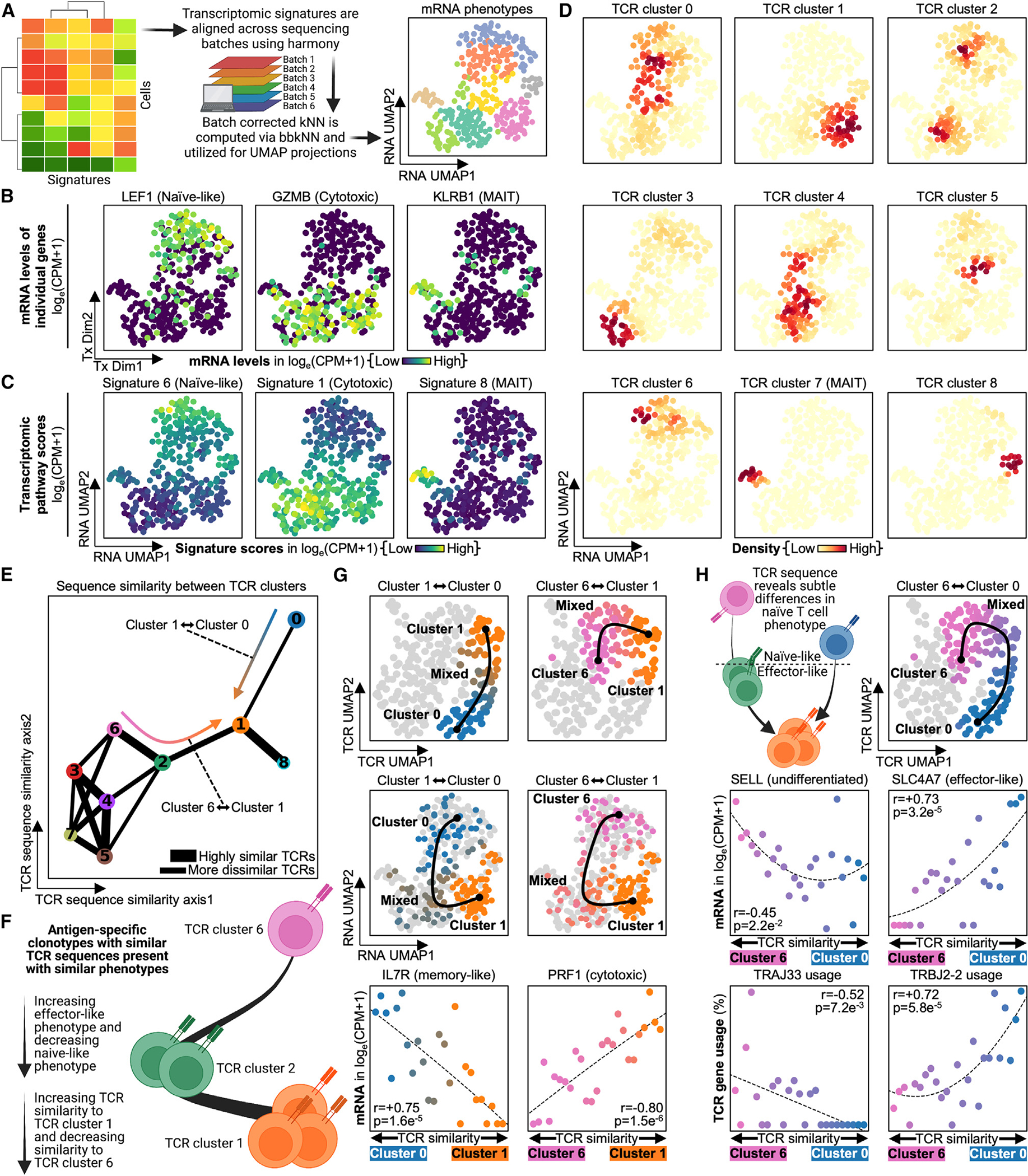
Phenotypic similarity between CD8^+^ T cell clonotypes targeting the same antigen is governed by TCR sequence similarity (A) Transcriptomic signatures are batch corrected via harmony and bbkNN to correct for technical differences from differing sequencing batches. Signatures are reduced to two dimensions via UMAP, and these transcriptomic dimensions are hereby called “RNA UMAP.” (B) Scatterplot of RNA UMAP with select mRNAs colored, with legend on the bottom. (C) Scatterplot of RNA UMAP with select signatures colored, with legend on the bottom. (D) Scatterplot of RNA UMAP with densities of each TCR cluster colored, with legend on the bottom. (E) Scatterplot of TCR-derived clusters with lines connecting the clusters by sequence similarity. The thicker the bars, the more similar the clusters are in terms of TCR sequence; similarity weaker than the thinnest line is not shown on the plot. Legend is on the bottom right. (F) Clonotypes are scored by their TCR sequence similarity for either TCR-derived cluster A or TCR-derived cluster B. This sequence similarity metric is correlated with phenotypic measurements. An example of this is given comparing sequence similarity to clusters 6 and 1 with naive- and effector-related transcripts. (G) Plots on the left compare sequences to TCR-derived clusters 1 and 0, and plots on the right compare sequences to clusters 1 and 6. Top: TCR sequence similarity on the TCR UMAP. Middle: TCR sequence similarity on the RNA UMAP. Bottom: scatterplot with TCR sequence similarity on the x axis, where positive means increasing similarity to one cluster and negative means increasing similarity to the other (see STAR Methods). y axis is the mRNA level for a given gene measured in log_e_(CPM+1). Dots are colored by the cluster they are most similar to in terms of TCR sequence: orange for TCR-derived cluster 1, pink for TCR-derived cluster 6, and blue for TCR-derived cluster 0. (H) Top left: TCR sequence similarity differences reveal subtle phenotypic differences between naive-like clusters 0 and 6. Top right: TCR sequence similarity comparing clusters 6 to 0 on the TCR UMAP. Middle: scatterplot with TCR sequence similarity on the x axis, where positive is increased similarity to cluster 0 and negative is increased similarity to cluster 6. y axis is the mRNA level for a given gene measured in log_e_(CPM+1). Bottom: scatterplot of TCR sequence similarity on the x axis, where positive is increased similarity to cluster 0 and negative is increased similarity to cluster 6. y axis is TCR gene usage. Dots are colored by the cluster they are most similar to in terms of TCR sequence: pink for TCR-derived cluster 6 and blue for TCR-derived cluster 0. Correlation coefficients and p values are labeled on respective plots.

We next asked, for T cells specific to SARS2-YLQ, whether cells with similar (but not identical) TCR gene sequences were also of similar phenotypes. In other words, did larger TCRα/β gene differences correlate with larger phenotypic differences? To investigate this, we first created a two-dimensional projection of antigen-specific CD8^+^ T cell transcriptomes (an RNA UMAP) to visualize the phenotypic differences between different clonotypes (Figure 2A; Table S2.1; see STAR Methods). For example, TCR cluster 1 largely occupies the cytotoxic region of the RNA UMAP, while TCR clusters 0 and 6 largely occupy the naive region (Figures 2B–2D, S3A, and S3B). Interestingly, we also observed that clusters close together on the TCR UMAP were also close together on the RNA UMAP (Figure S3C). As UMAP will cluster similar cells together, this suggests that T cell clonotypes that are closely related by TCRα/β sequence similarity are also related by phenotype similarity.

In order to quantitatively compare TCR sequence with phenotype, we quantified TCRα/β sequence similarity between clusters (Figure 2E; see STAR Methods). This metric confirmed that clusters similar in phenotype were also similar in TCR sequence. For example, TCR clusters 1 and 8, which are neighbors on the RNA UMAP, are also very similar in TCR sequence (note the thick edge connecting these two TCR clusters). On the other hand, TCR clusters 6 and 1, which represent more undifferentiated and more cytotoxic phenotypes, respectively, have quite different TCR sequences. This further suggests that clonotypes with similar TCRs also have similar phenotypes and raises the possibility that TCR sequence similarity alone is suggestive of T cell phenotype similarity. For example, we hypothesized that clonotypes with TCR sequences similar to TCR cluster 1, which had an effector-like phenotype, would present with transcriptomes that were also more cytotoxic, while clonotypes with sequences more similar to cluster 6, which was more naive-like, would be more undifferentiated (Figure 2F).

We then sought to test our hypothesis that clonotypes with elevated effector-like characteristics were more similar in sequence to TCR cluster 1 and more dissimilar to TCR clusters 0 and 6. Indeed, as clonotypes presented with TCR sequences more similar to TCR cluster 1, they not only moved closer to cluster 1 on the TCR UMAP but they also moved closer to cluster 1 on the transcriptomic UMAP (Figure 2G, top and middle). Further, clonotypes with increased sequence similarity to cluster 1 also had lower levels of memory-associated *IL7R* and cytotoxic-associated *PRF1* (Figure 2G, bottom; Tables S2.2 and S2.3).^35,36^ In addition, the *IFNG*-producing YLQ-specific clonotypes reported by Shomuradova et al. were more similar in sequence to cluster 1 than to sequences from clusters 0 and 6 (Figure S3D).^25^ Thus, we demonstrate, and verify through literature, that increased TCR sequence similarity to cluster 1 significantly associates with an increased effector-like phenotype and the upregulation of effector-related mRNA.

The ability to differentiate effector and non-effector phenotypes through TCR sequence similarity led us to question if we could differentiate even smaller phenotypic differences through sequence similarity. An example is TCR clusters 0 and 6, which are both relatively undifferentiated but also very distinct in their TCR sequences (Figure 2H, top; Table S2.4). In fact, sequence similarity analysis does appear to resolve significant phenotypic differences between these clusters. Cluster 6 was more undifferentiated-like with higher expression of *SELL*, while cluster 0 already showed signs of effector differentiation with increased levels of *SLC4A7*, which has been tied to effector-like CD8^+^ T cells (Figure 2H, middle).^37,38^ We further suggest that these phenotypic differences are underpinned by TCR structural differences, as these clusters utilize unique TCR genes, with Jβ gene TRBJ2–2 specific for cluster 0 and Jα gene TRAJ33 specific for cluster 6 (Figure 2H, bottom). Thus, we demonstrate that increases in TCR sequence similarity strongly correlate with increases in phenotypic similarity, and we show that sequence similarity alone can resolve both minute and grand differences in the phenotypes of antigen-specific CD8^+^ T cells that can, in part, be verified through literature.

### Antigen-specific CD8^+^ T cell persistence is influenced by TCR sequence similarity

**Figure 3.**
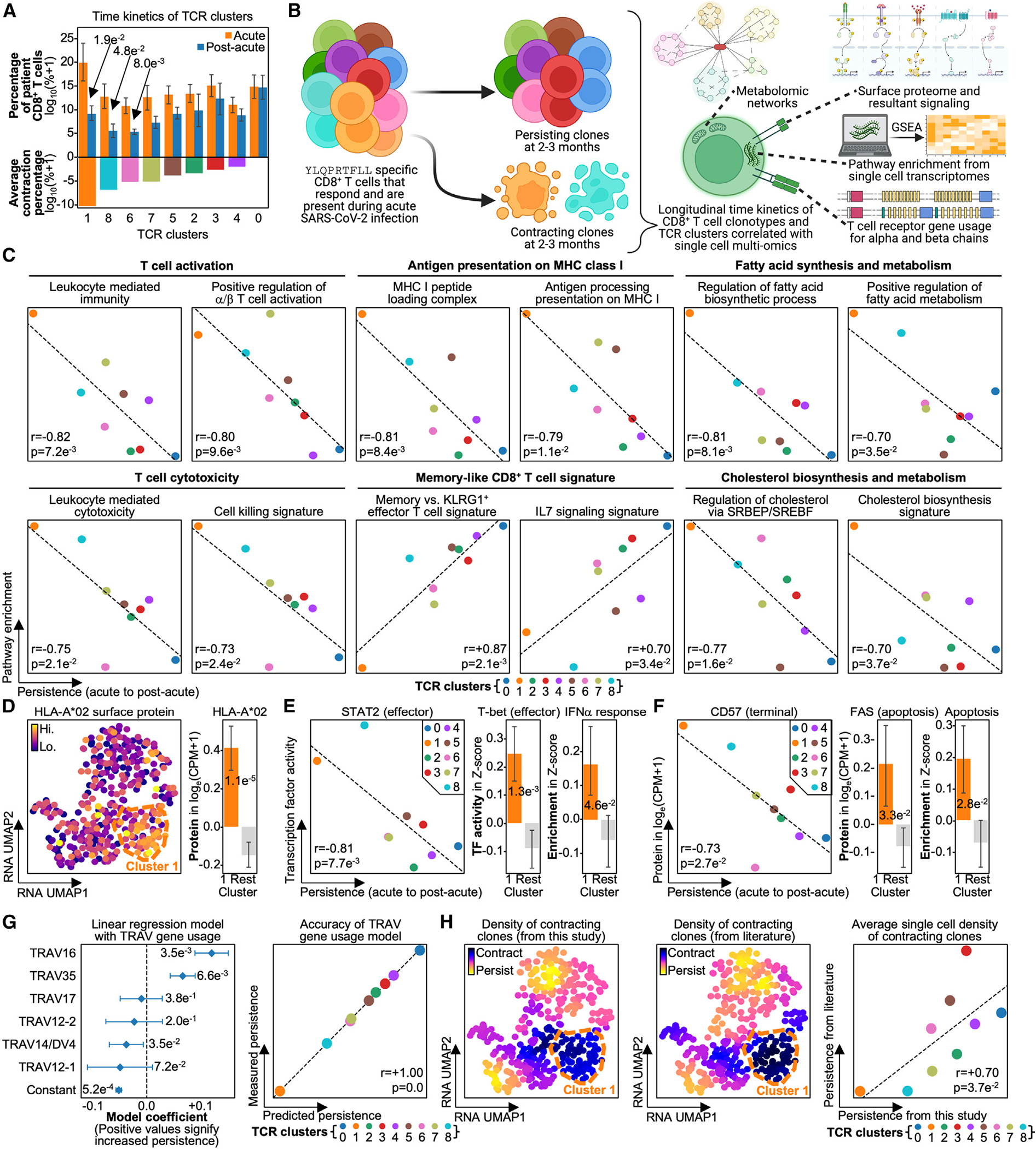
Persistence of CD8^+^ T cell clonotypes specific for the same antigen is correlated with TCR gene usage (A) Top: bar plot of TCR clusters on the x axis and percentage of a patient’s CD8^+^ TCR repertoire at the given time point on the y axis. Bar is colored by blood draw time point. Bottom: bar plot of TCR clusters on the x axis and average contraction percentage on the y axis. Bar is colored by TCR cluster. (B) Longitudinally tracked dynamics of YLQPRTFLL-specific CD8^+^ T cells are computed to identify persisting TCR clusters and contracting TCR clusters. Time kinetics are correlated with single-cell multi-omics features. (C) Scatterplot of TCR clusters with average contraction percentage on the x axis and pathway enrichment derived from gene set variation analysis (GSVA) on the y axis. Dots are colored by TCR cluster, linear fitted line is on plot, and T cell behavior and function are labeled in bold. (D) Left: scatterplot of RNA UMAP with HLA-A*02 surface protein levels colored. TCR cluster 1 is outlined in dashed orange. Middle: bar plot of TCR cluster 1 versus rest on the x axis and surface protein level on the y axis. Right: bar plot of TCR cluster 1 versus rest on the x axis and transcription factor (TF) activity on the y axis. (E) Left: scatterplot of TCR clusters with average contraction percentage on the x axis and TF activity on the y axis. Dots are colored by TCR cluster, linear fitted line is on plot, and the legend is in the top right. Middle: bar plot of TCR cluster 1 versus rest on the x axis and TF activity on the y axis. Right: bar plot of TCR cluster 1 versus rest on the x axis and pathway enrichment on the y axis. (F) Left: scatterplot of TCR clusters with average contraction percentage on the x axis and surface protein level on the y axis. Dots are colored by TCR cluster, linear fitted line is on plot, and the legend is in the top right. Middle: bar plot of TCR cluster 1 versus rest on the x axis and surface protein level on the y axis. Right: bar plot of TCR cluster 1 versus rest on the x axis and pathway enrichment on the y axis. (G) Left: coefficient plot of a linear regression model fitted on TCR α chain V gene usage per TCR cluster. Plotted confidence intervals are 95^th^ percentile, and means are represented by diamonds. Right: scatterplot of predicted versus true contraction, with the legend on the bottom. (H) Left: scatterplot of RNA UMAP with density of contracting clones as computed from this study colored. Middle: scatterplot of RNA UMAP with the color indicating the density of clones predicted to be contracting from literature curated antigen-TCR pairs. Right: scatterplot of density of contracting clones from this study on the x axis and clones predicted to be contracting on the y axis, with the legends on the bottom and on the plot. Significant p values are labeled on respective plots. Error bars on bar plots represent standard error, and the bar level represents the mean value of the given variable. Correlation coefficients and p values are labeled on respective plots.

Differences in antigen-specific CD8^+^ T cell phenotype have been well associated with differences in persistence after infection.^4,39–41^ Thus, we hypothesized that TCR sequence difference metrics might resolve differences in T cell persistence. Here, we define persistence as those clonotypes that persist at convalescence, 2–3 months after initial infection. To investigate this, we first compared the prevalence of each TCR cluster in patient blood from acute disease to convalescence. Indeed, the TCR clusters exhibited clear differences in the amount of contraction (Figure 3A; Table S3.1). While all clusters displayed some level of contraction, as expected of antigen-specific cells following viral clearance, TCR cluster 1 presented with the greatest level of contraction, followed by TCR cluster 8. As persistence is influenced by phenotype, we hypothesized that TCR cluster 1 may exhibit the most contraction because it is composed of short-lived effector cells (SLECs) during acute disease. This hypothesis is also consistent with the significant prevalence of TCR cluster 1 in patient blood during acute illness, as increased clonal expansion has been reported to occur in SLECs during early and acute infection stages. Subsequent contraction of those same cells at convalescence can protect the body from immunopathology.^4,42^

To confirm that cells from strongly contracting TCR cluster 1 were SLECs, we correlated persistence with phenotype (e.g., transcriptome, proteome, and metabolome from gene set enrichment analysis [GSEA]) (Figure 3B). Indeed, TCR cluster 1 presented with significantly increased T cell activation, cytotoxicity, fatty acid and cholesterol synthesis, and MHC class I presentation pathways, all of which have been reported as representative of SLECs (Figure 3C; Table S3.2).^42–44^ Increased MHC class I presentation, which is downstream of type I IFN signaling (a hallmark of SLECs), was confirmed through upregulation of HLA-A*02 surface protein levels; surface proteins were quantified via single-cell (sc)-CITE-seq (Figure 3D; Table S3.3).^45^ TCR cluster 1 also exhibited other features of type I IFN signaling, including type I IFN-induced transcription factors STAT2 and T-bet (Figure 3E; Table S3.4).^46–50^ The SLEC phenotype of TCR cluster 1 was also confirmed through the upregulation of the terminal differentiation protein CD57 and the death receptor FAS and the activation of apoptotic pathways (Figure 3F).^51–53^ Notably, TCR cluster 8, which closely followed TCR cluster 1 in its magnitude of contraction, displayed the second highest levels of these SLEC-related signatures (Figures 3C–3F), thus reinforcing the notion that SLEC-like phenotypes exhibit more contraction after infection. Notably, TCR clusters 1 and 8 are not just similar phenotypically, but they also exhibit a high degree of TCR sequence similarity, and they exhibit similar levels of persistence.

We further tested this connection between TCR sequence and persistence by correlating TCR gene usage with persistence. In fact, TCR gene usage and persistence were strongly correlated, especially for Vα genes (Table S3.5). This was underscored by the highly accurate ability of Vα gene usage to predict the persistence of each TCR-derived cluster in a linear regression model (Figure 3G; Table S3.6). Interestingly, Vα genes that conferred increased contraction, such as TRAV12–1 and TRAV12–2, were more similar in sequence than those that conferred persistence, such as TRAV16 and TRAV35 (Figure S4). This underscores the correlation between TCR sequence similarity and persistence, as TCR genes with similar CDR3 sequences confer similar impacts on clonotype persistence.

We sought to independently verify this relationship between TCR sequence and persistence by comparing our contracting clonotypes with those from Minervina et al., who also identified SARS2-YLQ-specific CD8^+^ T cell clonotypes that contracted 2–3 months post-infection.^8^ Using TCR sequence similarity, we utilized those reported contracting clonotypes to predict which of our clonotypes would contract. In fact, the contracting clonotypes from the literature and those identified from our study occupied similar regions on the RNA UMAP (Figure 3H, left and middle). This suggests that antigen-specific clonotypes that contract after acute disease share common TCR sequences and transcriptomes across multiple cohorts. This was quantitatively confirmed by the strong positive correlation between the amount each cluster contracted and the sequence similarity of each cluster to the literature-reported contracting clonotypes (Figure 3H, right). Thus, the antigen-specific T cell clonotypes that contract post-infection exhibit high sequence similarity across independent cohorts. This may suggest, for at least this particular immunogenic SARS-CoV-2 antigen, that a universal relationship exists between TCR sequence and persistence.

### TCR sequences are more important than environmental signals in influencing the phenotype of antigen-specific clonotypes

**Figure 4.**
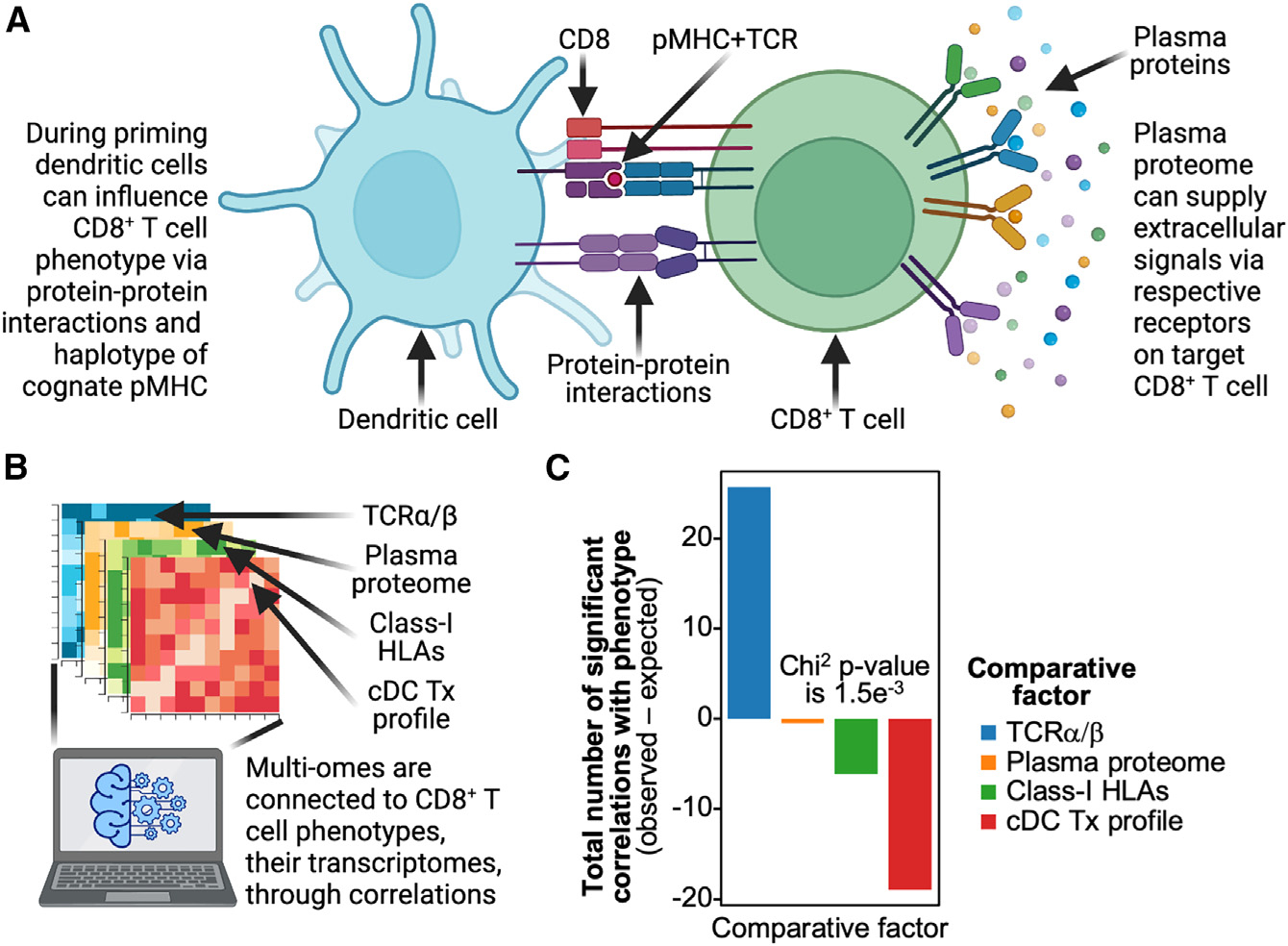
Phenotypes of CD8^+^ T cells responding to the active infection are primarily governed by TCRα/β sequences over other -omics (A) CD8^+^ T cells are influenced by their plasma protein environment, initial interaction with priming dendritic cell, and the other HLAs present in a patient. (B) These different -omics along with the TCRα/β -omics are correlated with transcriptomic phenotypes to identify the importance of each -omic. (C) Bar plot of each -omic on the x axis and the total number of observed minus expected significant correlations with unbiased PCA dimensions summed for each -omic on the y axis; negative bars indicate fewer significant correlations with T cell phenotype than statistically expected, while positive bars indicate more significant correlations with phenotype than expected. Significant p values are labeled on respective plots.

The strong associations between TCR sequences and T cell phenotype led us to question if TCR sequences were more influential than the environmental factors purported to affect T cell phenotype (Figure 4A). To investigate this, we compared the relative influence of TCR sequences on T cell phenotype with the environmental factors of plasma proteins, patient HLA-haplotype-specific protein-protein interactions, and conventional dendritic cell phenotypes. Plasma proteins can steer phenotype selection through cytokine signaling,^5^ while differences in patient HLA haplotype can correlate with phenotypic differences, possibly through interactions between MHCs and co-receptors on T cells.^6–8,11^ Our last comparator, the conventional dendritic cell (cDC) phenotype, can potentially influence T cell phenotype through mechanisms of cytokine and co-receptor signaling during priming.^9,10^ For this comparison, we quantified each factor into latent dimensions and correlated these dimensions with antigen-specific CD8^+^ T cell phenotypes (Figure 4B; Table S4; see STAR Methods). If these factors all influenced T cell phenotype equally, we would observe significant correlations splitting equally among the factors. Notably, we observed the opposite: TCR sequences had a much larger number of significant correlations with phenotype than any of the environmental factors (Figure 4C). Next, while the existence of MAIT cells as SARS2-YLQ specific has been demonstrated by previous studies,^25^ they could be confounding our analysis. However, even when they are removed from the dataset, we still observe the same striking phenomena of TCR sequences possessing the greatest number of significant correlations with phenotype compared with any environmental factor (Figure S4B). Thus, for antigen-specific CD8^+^ T clonotypes directly responding to SARS2-YLQ antigen, not only do TCR sequences strongly correlate with phenotype and persistence, but they appear to influence T cell phenotype much more strongly than do environmental factors.

To further validate our finding that TCR sequence strongly impacts T cell phenotype, we looked to our previously reported set of CD8^+^ T cell clonotypes experimentally validated to be specific for the immunogenic SARS-CoV-2 antigen RLITGRLQSL (RLIT).^54^ RLIT-specific clonotypes were derived by cloning RLIT-specific TCRs into primary CD8^+^ T cells from HLA-A*02:01 healthy donors. When we clustered RLIT-specific clonotypes by their TCR gene usage and sequence, we observed two distinct TCR clusters that directly corresponded to two distinct cytokine secretion patterns (Figure S4C). Cluster A TCRs were characterized by similar V-J gene usage and CDR3 sequence (Figure S4D) and displayed significantly decreased secretion of tumor necrosis factor α (TNFα), IFNγ, and granzyme B after exposure to RLIT-presenting antigen-presenting cells (APCs) (Figure S4E). Thus, we experimentally demonstrate that TCR sequence alone can indeed stratify and affect CD8^+^ T cell effector activity.

### Bystander T cell phenotype is still primarily influenced by TCR sequences

Our analysis of COVID-19 patients provided the unique opportunity to compare antigen-responding clonotypes with bystander T cells. During SARS-CoV-2 infection, CMV-specific cells are reported to be bystander activated, as they present with effector phenotypes despite a lack of CMV viremia, and Flu-specific cells are described as having no reactivation, as they present with memory-like phenotypes.^29,55,56^ Previous works in microbial infection contexts have shown that environmental cytokine signals can trigger bystander activation.^57^ However, these previous works did not thoroughly investigate the role of TCR sequences. Therefore, it is unclear if bystander T cell phenotypes, and thus bystander activation, is primarily influenced by TCR sequence, as we demonstrated for antigen-responding T cells, or if bystander T cell phenotype is dominantly determined by environmental factors.

**Figure 5.**
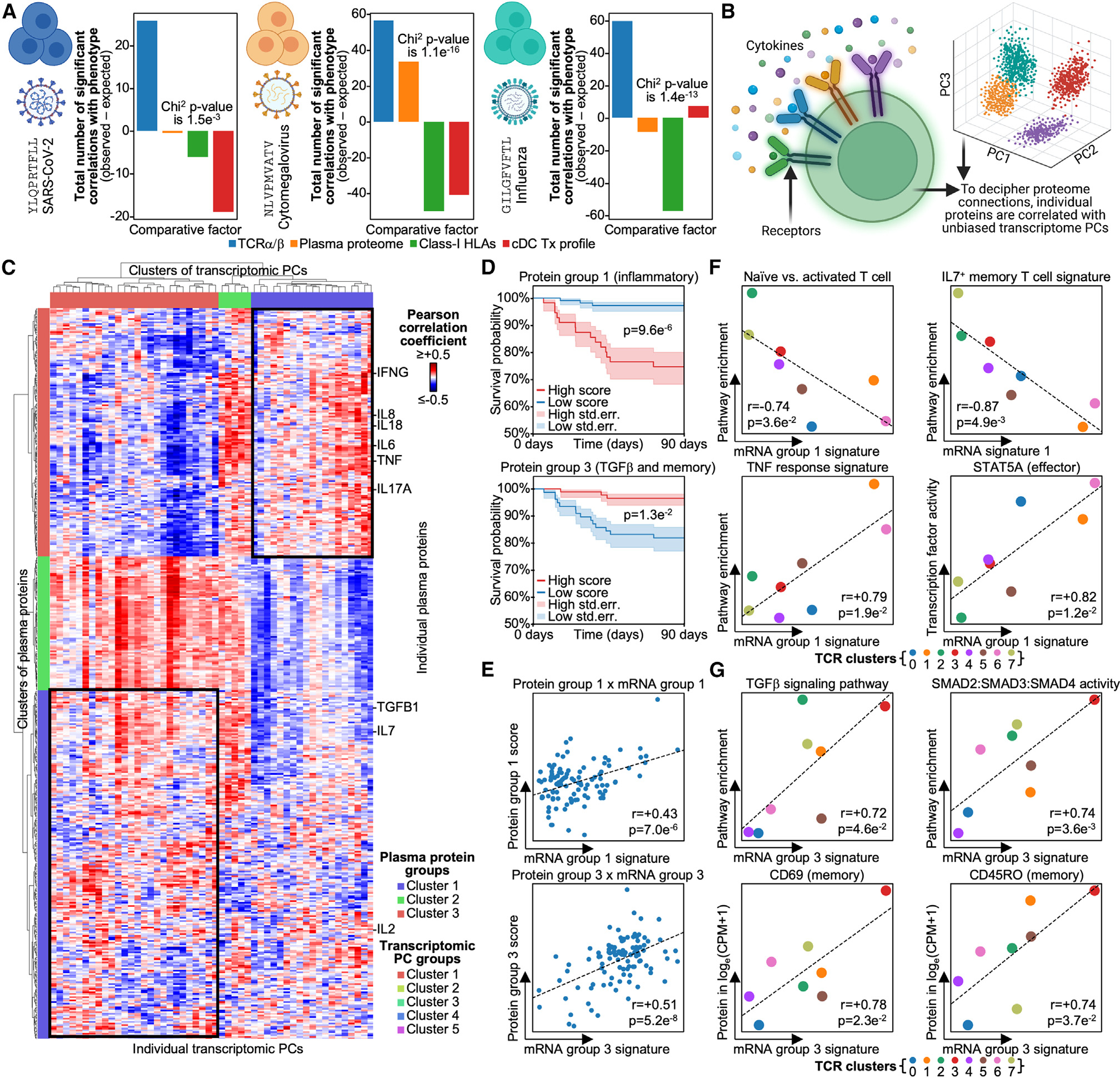
CMV-specific CD8^+^ T cells show considerable proteomic influence on their in-disease phenotype (A) Bar plot of each -omic on the x axis and the total number of observed minus expected significant correlations, with unbiased PCA dimensions summed for each -omic on the y axis for CD8^+^ T cells specific for YLQPRTFLL on the left, NLVPMVATV in the middle, and GILGFVFTL on the right. (B) To decipher the plasma proteins influencing CMV-NLV-specific CD8^+^ T cell phenotype, we correlate individual plasma proteins with unbiased PCA dimensions and then identify single-cell multi-omics features underpinning PCs with said plasma proteins. (C) Correlation matrix with rows as plasma proteins and columns as unbiased transcriptomic PCs from CMV-NLV-specific CD8^+^ T cells. The top annotation row is unbiased clustering of PCs, and the left annotation column is unbiased clustering of proteins. Black boxes denote areas of interest explored in the top box in (D) and the bottom box in (E). (D) Kaplan-Meier survival curve with shaded area as standard error. Patients are divided by high and low levels of the labeled protein group, with the legend on the bottom. (E) Scatterplot of mRNA score on the x axis and protein score on the y axis. The transcriptomic PC and protein groups being compared are labeled on the top. Dots are blood draws (per patient per time point), and linear fitted line is on plot. (F) Scatterplots of TCR clusters with mRNA signature of transcriptomic PC group 1 on the x axis and pathway enrichment or TF activity on the y axis. Dots are colored by TCR cluster, and linear fitted line is on plot, with the legend on the bottom. (G) Scatterplots of TCR clusters with mRNA signature of transcriptomic PC group 3 on the x axis and pathway enrichment or TF activity on the y axis. Dots are colored by TCR cluster, and linear fitted line is on plot, with the legend on the bottom. Significant p values are labeled on respective plots. Correlation coefficients and p values are labeled on respective plots.

To resolve this, we correlated TCR sequences and environmental factors with the measured T cell phenotypes for both bystander-activated CMV-NLV-specific T cells and non-reactivated, Flu-GIL-specific T cells. Strikingly, TCR sequences were more significantly associated with phenotype than any environmental factor for both sets of bystanders (Figure 5A). This further accentuates the importance of TCR sequence on phenotype even in the context of bystander activation and suggests that the strong influence of TCR sequence on phenotype may be a more universal phenomenon due to its occurrence for multiple antigen-specific populations.

### Inflammatory cytokine signals play significantly stronger roles in bystander activation than SARS-CoV-2-specific CD8^+^ T cells

Notably, plasma proteins exhibited significant correlations with the phenotype of CMV-NLV-specific CD8^+^ T cells. Existing literature points to certain inflammatory proteins, such as IL-18, as leading to bystander activation in microbial infection contexts. Thus, we sought to identify the specific cytokines that correlated with phenotype in our bystander-activated cells (Figure 5B).^5,58,59^ In agreement with previous reports, when we correlated plasma proteins with a transcriptomic phenotype, we similarly resolved a group of inflammation-related cytokines, namely IL-6, IL-18, and TNF (Figure 5C; Table S5.1).^60^ We further resolved a group of plasma proteins that are either anti-inflammatory or memory T cell associated, namely transforming growth factor β (TGF-β), IL-2, and IL-7.^35,61–63^ These two protein groups correlated with two distinct transcriptomic states. Importantly, patients with high levels of each group exhibited divergent survival fates, with the inflammatory group correlating with decreased survival and the anti-inflammatory group correlating with increased survival (Figure 5D). The inflammatory protein group exhibited a positive correlation with mortality, which likely represents known relationships between inflammation and disease severity in COVID-19 patients.^17,60^ The survival associations of these proteins suggests that the two different CMV-specific cell phenotypes they correlate with are also associated with patient survival. Thus, bystander-activated CMV-specific cells consist of two distinct phenotypes associated with two divergent cytokine environments and opposing patient mortality rates.

To investigate what underpins the differences between these two phenotypes, we next investigated what transcriptomic signatures in CMV-NLV-specific cells correlated with each protein group (Figure 5E; see STAR Methods). Inflammatory proteins strongly associated with effector phenotypes, while the TGF-β protein group was correlated with more quiescent and memory-like cells (Figures 5F and 5G; Tables S5.2–S5.5). For example, bystander-activated cells in inflammatory cytokine environments displayed decreased naive and memory signatures paired with an increase in TNF responses genes and STAT5A activity. Interestingly, STAT5A is known to maintain effector responses and so may contribute to the effector phenotype of bystander-activated CMV-specific cells.^49,64,65^ In contrast, bystander-activated cells in the TGF-β and memory plasma protein environment upregulated memory-associated proteins CD45RO and CD69 along with TGF-β response and SMAD signaling genes.^66,67^ TGF-β signaling and its downstream transcription factor SMAD have been reported to suppress effector T cell function, which may explain why these bystander-activated cells display more memory-like features.^66–68^

Even though these two CMV-NLV-specific bystander-activated phenotypes display strong correlations with environmental cytokine signals, the phenotype of these cells was still primarily explained by TCR sequence alone (Figure 5A, middle). Indeed, when we compare the TCR sequences of these two phenotypes, we observed marked differences in their TCR α- and β-chain gene usage and motifs (Figures S5A and S5B). For example, effector-like bystander-activated clonotypes presented with a “CAASGGQNFVF” TCRα motif, while more memory-like clonotypes utilized a “CIMNTGFQKLVF” motif. Thus, we demonstrate that TCR sequences are the most influential factor on phenotype for both antigen-responding and bystander CD8^+^ T cells for at least the four immunogenic antigens explored here. Further, we demonstrate that even in the context of bystander activation, where cytokine signals play significant roles, TCR sequences continue to dominate as the strongest contributor to antigen-specific CD8^+^ T cell phenotype.

## DISCUSSION

The factors underpinning antigen-specific CD8^+^ T cell phenotype and persistence have been long sought after due to their fundamental relevance for understanding immunity against infections and cancers and as metrics for guiding T cell-related therapy and vaccine design. Previous works have established that TCR sequence, cytokines, and co-stimulatory proteins are factors central to T cell phenotype and persistence.^3,4,6,11,30,41,69–72^ However, these efforts have only qualitatively examined these phenotype-influencing factors in isolation from each other, typically within murine models or cell lines, and are often uncontrolled for cognate antigen or MHC. This substantially limits the applicability of such findings to the context of antigen-specific cell responses in human disease. For example, Schattgen et al. identified a novel CD8^+^ T cell population that displayed a unique correlation between TRBV30 gene usage and *EPHB6* mRNA levels; however, their analyses were centered on peripheral blood mononuclear cells (PBMCs) from a few healthy donors.^30^ In other relevant work, Lagattuta et al. revealed how TCR sequences influence CD4^+^ T cell fate and demonstrated how TRBV gene usage explains which cells differentiate into regulatory T cells (Tregs); however, their analyses did not resolve antigen specificity.^3^

Here, we explored quantitative comparisons of CD8^+^ T cell phenotype-influencing factors in a large HLA-haplotype-matched patient cohort in a specific disease setting. We comprehensively investigated the factors that predetermine the phenotype and persistence for both antigen-dependent activation as well as in bystander-activated and non-reactivated CD8^+^ T cells from a longitudinal cohort of 68 COVID-19 patients reflecting the full range of infection severities. Each cell was deeply phenotyped through paired clinical measures, plasma multi-omics (454 proteins), HLA haplotype, and simultaneous measurement of TCR sequences and multi-omics single-cell phenotyping (covering 679 antigen-specific clonotypes gathered from 171,696 single CD8^+^ T cells). For four immunogenic antigens representing three different viruses, we reveal that TCR sequences are, by far, the most important factor influencing both phenotype and long-term persistence of antigen-specific and bystander-activated and non-reactivated CD8^+^ T cells during and after SARS-CoV-2 infection.

We also find that the persistence of SARS-CoV-2-specific cells months after infection is predetermined by TCR gene usage, indicating that TCR sequence determines the long-term future fate of antigen-specific T cells. Previous literature has associated memory-precursor effector cells (MPECs) with decreased effector activity and seemingly immediate upregulation of memory markers during initial infection.^41,42,73^ For example, the measured persistence of TCR clonotypes in TCR cluster 0 from acute disease to convalescence aligns with strong memory signatures in their transcriptomes. Thus, their persistence may be explained by their acquirement of a memory pre-cursor phenotype that uniquely associates with their TCR sequences and gene usages. In contrast to MPECs, SLECs are defined through their apoptotic fate, which is associated with an upregulation of terminal differentiation and effector-related proteins.^41,42,73^ We reveal that this difference in phenotype between MPECs and SLECs is actually underpinned by intrinsic TCR sequence. This suggests, given a specific antigen, that it is TCR sequence that primarily influences which antigen-specific cells go on to form long-lived memory several months after infection. One possibility is that the nature of T cell activation is controlled through allosteric signaling effects, which are, in turn, mediated by TCR gene usage. This suggests that future studies focused on the structure and dynamics of TCR-antigen-MHC complexes may help resolve some mechanistic insights into the TCR gene-T cell phenotype relationship.^74^

While TCR sequences have been a central target of antigen-specific T cell research, the role of the TCRα/β genes in bystander activation has received little attention, as bystander activation has been primarily attributed to cytokine signaling leading to the formation of activated T cell phenotypes.^5,57,75–77^ While we find, surprisingly, that TCR sequences are the primary factor that explains the phenotype of bystander-activated cells in COVID-19 patients, we also confirm that inflammatory cytokines such as IL-18 have a positive association with bystander-induced effector phenotype. The underpinning mechanism that explains how TCR sequences contribute to the phenotype of bystander-activated cells warrants further investigation. One possibility is that prior to reactivation of a given cell, TCR gene use may anticipate the available epigenetic landscape. This landscape may shape responses to stimulatory cytokine signals in shaping the phenotype of bystander-activated cells.

The results presented here are reminiscent of a prevalent theory in literature called “original antigenic sin.”^78^ In the context of T cells, this theory suggests that a T cell’s initial encounter with antigen determines its phenotype for the duration. This theory has been demonstrated in multiple contexts such as dengue fever.^79,80^ Our study suggests that, given a specific antigen-MHC, TCR sequences dominantly influence the phenotype of antigen-specific cells. Thus, “original antigenic sin” appears to actually predate the antigen encounter, at least for the specific immunogenic antigens explored here.

### Limitations of the study

This study only examines CD8^+^ T cells specific for one of four immunogenic viral antigens presented by a single HLA allele. While this HLA allele and these antigens are common in humans, it does serve as a limitation and suggests that true generalization of these findings will require the generation and analysis of datasets similarly deep to ours for T cell responses to different antigens on diverse HLA alleles. In addition, we do note that while our cohort demonstrates substantial variation in the different -omics, such as plasma proteome and cDC transcriptome, there are features of each space that are distinct between infection states. Further, although we resolve similarly strong overall T cell clonotype-T cell phenotype relationships for all four antigen specificities explored here, the details of those relationships are highly antigen dependent. It will likely take a substantially larger dataset of well-characterized T cell clonotypes with specificities to large numbers of diverse antigens presented by multiple HLA alleles to extract more general rules. However, emerging technologies should make such datasets available in the near future. Thus, while these limitations highlight important notes to consider, they do not detract from the importance and relevance of the relationship we reveal here between TCR sequence and phenotype.

## STAR★METHODS

### RESOURCE AVAILABILITY

#### Lead contact

Further information and requests for resources and reagents should be directed to and will be fulfilled by the lead contact, James R. Heath (jim.heath@isbscience.org).

#### Materials availability

This study did not generate new unique reagents.

#### Data and code availability

- pMHC-TCR pairings were downloaded from publicly accessible databases (VDJdb, IEDB, McPAS-TCR) and literature works.^54^ Single-cell data was downloaded from E-MTAB-10129 and https://data.mendeley.com/datasets/96v329bg7g/1.^56^ TCR sequences for contracting clones from day 15 to day 85 after acute SARS-CoV-2 infection were downloaded from https://github.com/pogorely/Minervina_COVID/.^8^ TCR sequences for *IFNG*-producing YLQ-specific CD8^+^ T cells were from Shomuradova et al., 2020.^25^.
- This paper does not report original code. Scripts were run using public Python and R packages and are available upon reasonable request.
- Any additional information required to reanalyze the data reported in this work paper is available from the lead contact upon request.

### EXPERIMENTAL MODEL AND STUDY PARTICIPANT DETAILS

The INCOV cohort included 209 SARS-CoV-2 patients (50% females, aged between 18 and 89 years with an average of 56 years); this cohort was introduced in our previous manuscripts.^16,93^ Participants were identified at five hospitals of Swedish Medical Center and affiliated clinics located in the Puget Sound region near Seattle, WA. All enrolled patients provided written in-person informed consent and was obtained in our previous aforementioned studies. Procedures for the INCOV study were approved by the Institutional Review Board (IRB) at Providence St. Joseph Health with IRB study number STUDY2020000175 and the Western Institutional Review Board (WIRB) with IRB study number 20170658.

### METHOD DETAILS

#### GLIPH2 analysis for HLA-A*02:01 TCRs from public databases and literature

GLIPH2 was run on http://50.255.35.37:8080/ using the GLIPH2 algorithm, version 1.0 reference for CD8, with all_aa_interchange-able set to YES.^18^ pMHC-TCR pairs from public databases and literature were used if they contained paired TCR sequences and bound to HLA-A*02:01. IEDB pairs were further filtered to only include valid TCR sequences, as in those without “#”. VDJdb was filtered for those pairs with a confidence of at least one. Antigen-specific CD8^+^ T cells were identified as those that belonged to the same GLIPH group of the target antigens (e.g., YLQPRTFLL, NLVPMVATV, or GILGFVFTL).

#### TCR clustering and projection

TCRdist from the CONGA package was used to compute TCR distances between YLQ-specific clonotypes using paired chain gene usage and amino acid sequences.^30^ This distance matrix was then reduced via PCA to create 50 PC dimensions. The two-dimensional UMAP projection was calculated via a 30 n-neighbor 20 n-PCs kNN graph and the UMAP algorithm.^81^ TCR clusters were computed via leiden with a resolution of 1.38, highest resolution in 0.01 increments that did not lead to over-clustering.^82^ CMV and GIL specific TCRs underwent the same analytic pipeline with slight adjustments the values of each parameter. CMV-specific TCRs were reduced via a 30 n-neighbors 50 n-PCs kNN graph and TCR clusters were computed via leiden with a resolution of 1.32. GIL-specific TCRs were reduced via a 30 n-neighbors 50 n-PCs kNN graph and TCR clusters were computed via leiden with a resolution of 1.51.

#### TCR cluster differential expression gene and signature analysis

Differentially expressed genes were called between clusters via sc.tl.rank_genes_groups through the Scanpy package using the “Wilcoxon” method which implements the Mann-Whitney U test.^83^ The top 25 differentially expressed genes were correlated with each other via pearson correlation implemented through scipy. The correlation matrix was clustered using “Wald’s” method. N-clusters from 10 through 20 were searched and the number of clusters with the highest silhouette score was utilized; this was n-clusters = 12. Function of each cluster was annotated through the individual genes in each cluster and enrichment of different pathways computed via Enrichr.^84^ “sc” stands for “scanpy”.

#### Generation of RNA UMAP from transcriptomic signatures

To derive single cell signature scores, we averaged the expression, in log_e_(CPM+1), of the genes per signature. This matrix of cell by signature was inputted into traditional single cell processing methods as a PC-like object. It was first batch corrected via harmony until convergence then a batch-corrected UMAP was computed using bbkNN and UMAP.^85,86^

#### TCR sequence similarity analysis

TCR sequence similarity analysis was conducted by first smoothing clonotype-clonotype transitions using diffusion map components (DCs). 15 DCs were computed based on the original TCR-derived kNN. These DCs were used to compute a new kNN with 20 n-neighbors. This new KNN was used to compute a PAGA network which was used to visualize sequence similarity. A sequence similarity metric was then calculated by taking the original 15 DCs and inputting them into Scanpy’s sc.tl.dpt pseudotime calculation. To achieve the TCR sequence similarity metric that compared the sequence similarity of a given clonotype to two TCR-derived clusters of interest we utilized the mentioned pseudotime calculation by averaging the pseudotime values from setting each cell within a given cluster as the root, this was repeated for the second cluster. These two pseudotime values were then subtracted from each other such that the above 0 values were biased toward TCR-derived cluster A and below 0 values were biased toward TCR-derived cluster B. Thus, a clonotype with a value of 0.5 would be more similar in TCR sequence to TCR-derived cluster A. When plotting scatterplots, we bin the x axis into 25 bins and use these bins to compare sequence similarity with a given variable.

#### TCR cluster contraction and correlation analyses

Percentages of each clonotype was calculated by dividing the number of cells attributed to a patient for a given timepoint by the total number of non-doublet high-quality CD8^+^ T cells for the same patient at the same timepoint. This percentage was then averaged per TCR cluster and compared via a t test without assuming equal variance. T test was used instead of Mann-Whitney U test because we were comparing percentages not transcriptomic data, the latter tends to have a negative binomial distribution.^87^ Contraction strength was equal to the differences in percentage (transformed via log_10_(%+1)) between convalescent (also called T3, post-acute) and acute disease (also called T1 or T2, early) timepoints. This contraction metric per cluster was then correlated with pathway enrichments computed from single cell GSVA scores, TF activity computed from pySCENIC, and surface protein levels as cells were assayed via CITE-seq.^88–90^ CITE-seq data was normalized as previously described by normalizing cells relative to background antibodies (via Z-scores) which consisted of low-expressed non-T-cell expressing antibodies (e.g., isotypes).^91^ These comparative metrics were all measured at T1 and T2.

#### TCR gene usage linear regression modeling

The TCR gene usage percentages of each TCR cluster were correlated with contraction strength. As TRAV had a large number of significant correlations, it was utilized for linear regression analysis. We utilized the statsmodels in Python to model contraction strength (persistence). The inputs to the linear regression model was TRAV gene usage (i.e., each clonotype had a column for each significant TRAV gene where the value is its percent usage). A constant term was added to determine the intercept of the model and account for inherent contraction and persistence dynamics known to occur after acute infection.^92^ This analysis provided p values to determine significant coefficients, and the intercept represented the typical contraction clusters underwent through time.

#### Comparison of contraction strength with literature defined contracting clones

Contracting clones from literature were compared to contracting clones identified in this study using TCR similarity analysis.^8^ The literature-retrieved clones were compared to this study’s TCRs using TCRα sequence as TCRα showed the strongest correlation with contraction behavior (Table S3.5). Density for contracting clones was computed as mentioned above. Predicted contraction is a min-max scaled value of the number of neighbors between each TCR in this study with literature reported contracting clones. The number of neighbors was determined as the number of contracting TCRs in literature that had a TCR distance less than the average distance between TCRs in this study. The prevalence of each TRAV gene was simply calculated by computing the percent of TCRs in the literature reported dataset that was annotated with the given TRAV gene.

#### Environmental factor importance and correlation analysis

Each environmental -omic was subject to an auto-encoder model so that 50 latent dimensions could be produced for each -omic. This auto-encoder model consisted of the original -omic in its native level of information (e.g., per clonotype for TCRs) then a hidden layer of 200 nodes, 100 nodes, 50 latent dimensions, 100 nodes, 200 nodes, then the original -omic. The model had a max n-iterations of 25 with ReLU activation and an “adam” solver. All -omics had loss that surpassed the elbow point of a loss curve suggesting that they at least approached if not nearly reached their minima, some models reached their minima and finished modeling before the max n-iterations. It is important note that it is can be difficult to surmise if a model has reached a local or global minima. These dimensions were then correlated with antigen-specific T cell transcriptomes. For YLQ-specific cells, this meant both unbiased PC dimensions and transcriptomic signatures. All other tested antigens were only compared with unbiased PC dimensions. The expected count for an individual dimension was defined as the number of tested variables (n-signatures or n-PCs) multiplied by the alpha used for significance which was 0.05 for this study. The expected value for the Chi^2^ test was 25% of the total as the null hypothesis is that all -omics have either random or equal importances with regards to phenotype. The heatmaps presented in the figures were clustered via “Wald’s” algorithm. Transcriptomic features were clustered across all -omics.

#### Interrogation of variance capture for correlated and compared -omics

It is important to note that we are looking at COVID-19 patients, and thus there are concerns as to whether each -omic is capturing the full possible variance of the given -omic space. For example, one may be concerned about whether we are only examining subtle difference in patient cytokine profiles (plasma proteins) as all COVID-19 patients may possess highly inflammatory profiles. This however is not true, rather, as detailed in our previous works,^93^ COVID-19 patients display strong variance in their captured plasma proteomes that not only varies more than a broad group of healthy donors in PC analysis, but also vary enough that patient disease severity can be distinguished from plasma proteome samples alone. We can ask this question for the conventional dendritic cell (cDC) -omic and find a similar answer that we capture much of the full variation space for cDCs. We not only capture cDCs as a whole, but our dataset also identifies rare dendritic cell phenotypes such as AS DCs (defined as AXL^+^SIGLEC6^+^) and distinguishes pro- and non-inflammatory CD1C^+^ cDC subtypes; all of which were only recently discovered.^16,94^ Lastly, we interrogate the HLA -omic for which these proteins can present allele-specific epitopes that activate and bind T cell co-receptors. Within the 68 HLA-A*02:01 patients we observe 43 different other HLA-A alleles, 78 different HLA-B alleles, and 39 different HLA-C alleles. Many of these alleles have been reported to bind to KIRs, one co-receptor that can bind HLA epitopes.^95,96^ None of these are trivial numbers and, considering we are examining 68 patients, represent incredible heterogeneity in the HLA genomic space. Thus, COVID-19 patients due capture the near, if not, full spaces of each of these -omics and present with substantial variance that allows these -omics to be compared with each other and TCR sequences in the comparative correlation analysis described above.

#### Plasma protein correlations with CMV-specific CD8^+^ T cell transcriptomic PCs

Unbiased transcriptomic PCs computed via a PCA analysis of single cell CMV-specific CD8^+^ T cell transcriptomes were correlated with individual plasma protein levels. This correlation matrix was clustered via “Wald’s” algorithm. N-clusters were searched via the same methods as the correlation matrix in Figure 1E by searching from n-clusters of 3–10 and determining the best n-clusters via the silhouette score of each clustering. The significantly correlated proteins or transcriptomic PCs for studied intersections were used to derive a protein and mRNA score, respectively. These protein scores were divided at a score of 0.7 for protein cluster 3 and 2 for protein cluster 1 to create high and low scoring patients at the T2 timepoint. These patient groupings were then used for survival analysis via a Kaplan-Meier curve and Chi^2^ test on a contingency table between overall survival and protein cluster score group assignment. The mRNA score was then correlated with GSVA, transcription factory activity, and surface protein values in a similar fashion to what was done between contraction strength and single YLQ-specific cell multi-omes. CMV-specific TCR clusters were computed in the same fashion as YLQ-specific TCRs using a leiden resolution of 1.32, again using the same method of finding the highest resolution that still avoids over-clustering clonotypes.

### QUANTIFICATION AND STATISTICAL ANALYSIS

#### Density calculation for TCR clusters and all other metrics

Densities for each TCR cluster could then be projected onto this UMAP by matching the TCR sequence of each single CD8^+^ T cell to the TCR cluster that sequence belongs to. Embedding density was first calculated via sc.tl.embedding_density then a 5 n-neighbor kNN graph was used to diffuse the values via five iterations to create a whole UMAP score for the density scores. All other metrics that required density calculations were implemented this same methodology.

#### Statistical analyses

All correlations were calculated using Pearson correlation, and all p values were calculated using Mann-Whitney U test unless otherwise specified. Bar charts were provided with error bars when multiple values were present, and these bars represented standard errors. Bar level represented the mean variable value. Fitted lines represent linear fitted lines via numpy.polynomial.Polyomial.

## Supplementary Material

1

2

3

4

5

6

## Figures and Tables

**KEY RESOURCES TABLE:** 

REAGENT or RESOURCE	SOURCE	IDENTIFIER

Deposited data		

COVID-19 patient scCITE-seq and scTCR- seq data (from same single cell)	Suetal.^16,17^	Array Express: E-MTAB-10129 ArrayExpress: E-MTAB-9357
VDJdb TCR-pMHC pairs	Goncharov et al.^19^	https://vdjdb.cdr3.net/
IEDB TCR-pMHC pairs	Vita et al.^21^	https://www.iedb.org/
McPAS TCR-pMHC pairs	Tickotsky et al.^20^	http://friedmanlab.weizmann.ac.il/McPAS-TCR/

Other		

Scanpy (v1.9.3)	Wolf et al.^84^	https://github.com/scverse/scanpy
CoNGA (v0.1.1)	Schattgen et al.^30^	https://github.com/phbradley/conga
pySCENIC (vO.12.0)	Van de Sande et al.^88^	https://github.com/aertslab/pySCENIC
GSVA (V1.38.2)	Hanzelmann et al.^91^	https://github.com/rcastelo/GSVA
harmonypy (v0.0.9)	Korsunsky et al.^86^	https://github.com/slowkow/harmonypy
bbkNN (v1.5.1)	Polanski et al.^87^	https://github.com/Teichlab/bbknn
GLIPH (v2)	Huang et al.^97^	http://50.255.35.37:8080/
UMAP (v0.5.3)	McInnes et al.^81^	https://github.com/lmcinnes/umap
Leiden (v0.9.1)	Traag et al.^82^	https://github.com/vtraag/leidenalg

## Highlights

- TCR sequence similarity correlates with CD8^+^ T cell phenotype similarity
- Antigen-specific CD8^+^ T cell behavior and persistence associates with TCR sequence
- TCR sequences have a greater impact than environmental signals on phenotype
- Even bystander T cell phenotype is most affected by TCR sequence, not environment

## INCLUSION AND DIVERSITY

We support inclusive, diverse, and equitable conduct of research.
