# T cell receptor sequences are the dominant factor contributing to the phenotype of CD8^+^ T cells with specificities against immunogenic viral antigens

**DOI:** 10.1016/j.celrep.2024.113841

**Published:** 2024-02-10

**Authors:** Daniel G. Chen, Jingyi Xie, Yapeng Su, James R. Heath

As this article was originally published on October 25, 2023, the legend depicted in Figure 5C entitled “Transcriptomic PC groups” listed five groups (clusters 1–5), each with color codes. This was a mistake; there are only three clusters in the figure, and the legend should have just included clusters 1–3. This error was accidental and did not affect any of the scientific results or conclusions of the paper. The paper has since been updated to reflect the correct figure. For readers’ reference, both the original and corrected figures appear below. The authors regret this error.


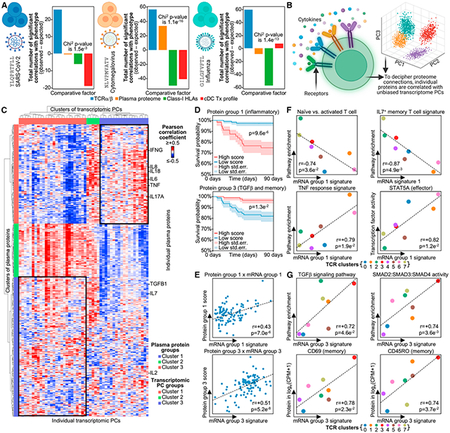


Figure 5. CMV-specific CD8^+^ T cells show considerable proteomic influence on their in-disease phenotype (corrected)


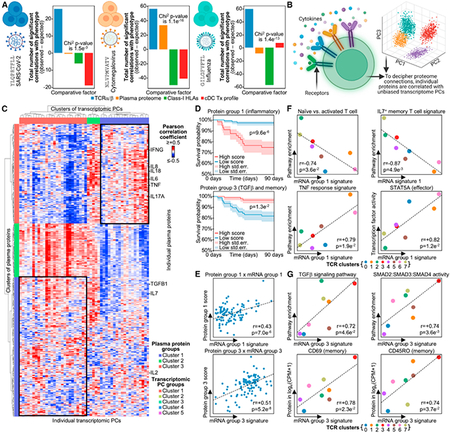


Figure 5. CMV-specific CD8^+^ T cells show considerable proteomic influence on their in-disease phenotype (original)

