# Supplementary material for: T cell receptor sequences are the dominant factor contributing to the phenotype of CD8+ T cells with specificities against immunogenic viral antigens
Source: Cell Rep. Author manuscript; Available in PMC 2023 Dec 19. (PMC10729740; doi:10.1016/j.celrep.2023.113279)
Supplement: 1 [file NIHMS1948172-supplement-1.pdf]

Cell Reports, Volume *42*

Supplemental information

T cell receptor sequences are the dominant factor contributing to the phenotype of CD8^{+} T cells with specificities against immunogenic viral antigens

Daniel G. Chen, Jingyi Xie, Yapeng Su, and James R. Heath

Supplementary figures

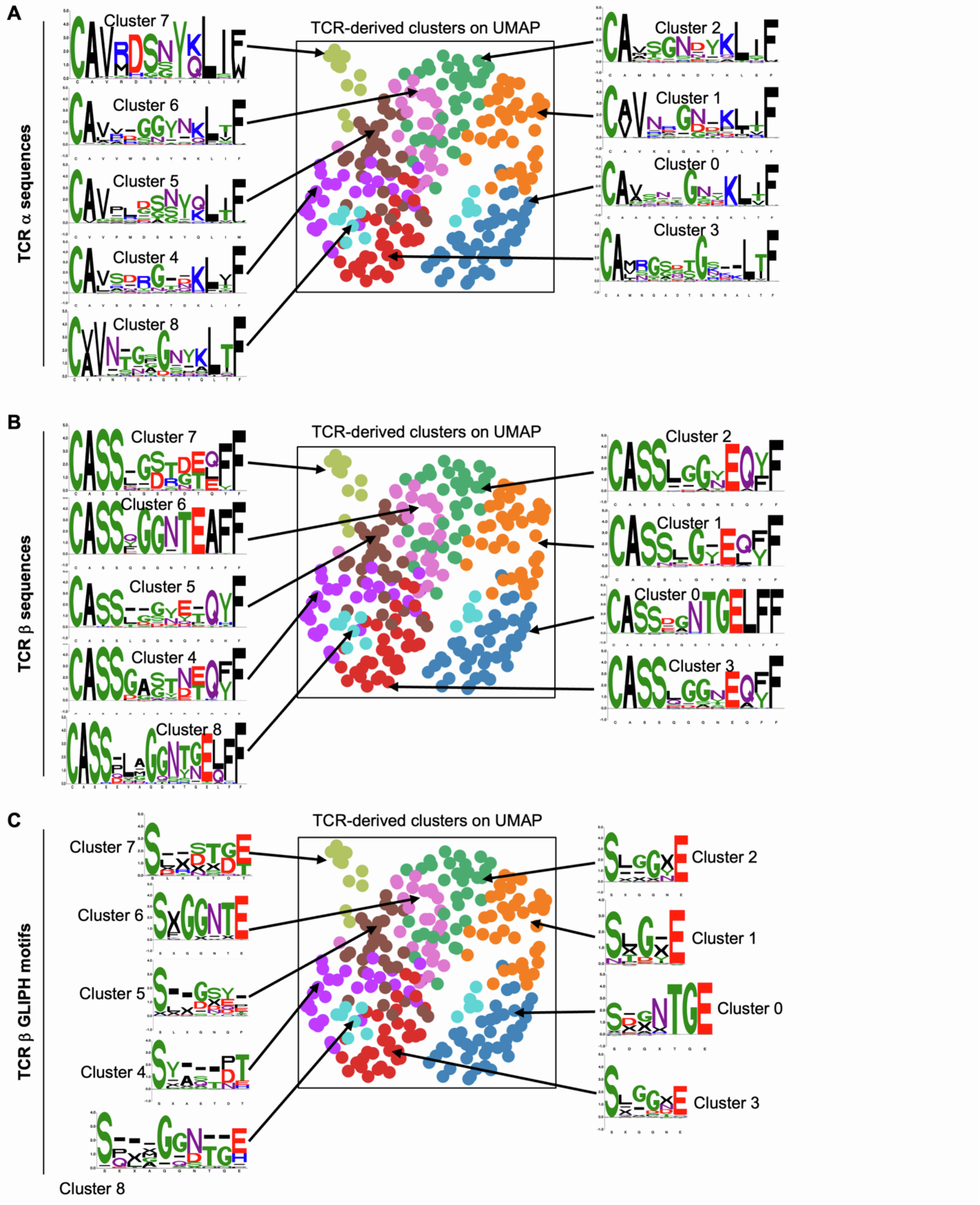

Supplementary Figure 1

Figure S1 (related to Figure 1): TCR clusters of YLQ-specific CD8$^+$ T cell clonotypes distinguished by unique TCR α and β sequences and GLIPH motifs

(A) TCR α motif plots for each cluster. TCR clusters are colored on the TCR-derived UMAP at the center of the plot with arrows connecting each motif plot to their respective location on the UMAP.

(B) TCR β motif plots for each cluster. TCR clusters are colored on the TCR-derived UMAP at the center of the plot with arrows connecting each motif plot to their respective location on the UMAP.

(C) GLIPH motif plots for each cluster. TCR clusters are colored on the TCR-derived UMAP at the center of the plot with arrows connecting each motif plot to their respective location on the UMAP.

A

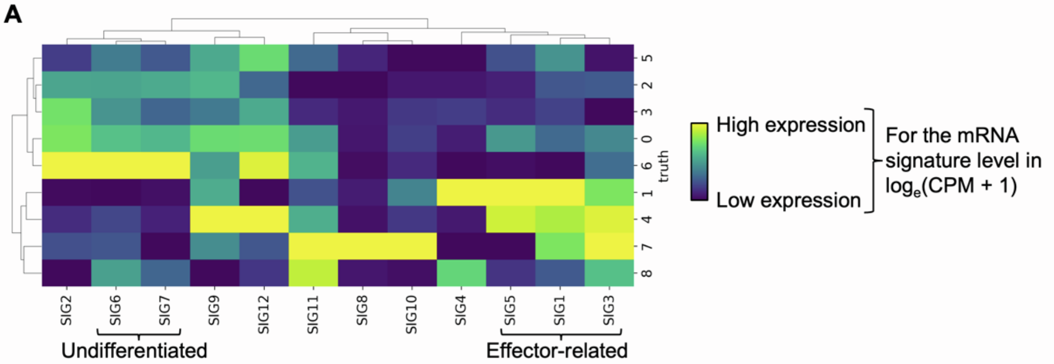

B

Shomuradova et al., 2020
Public *IFNG*-producing TCRs (C2)

Donor	TRBV	TRBJ	Sequence
1445	V6-4	J2-2	CASSYSDTGELFF
1445	V6-2	J2-2	CASSYSDTGELFF
1445	V10-2	J2-2	CASSYSDTGELFF
1445	V6-6	J2-2	CASSYSDTGELFF
1445	V6-6	J1-1	CASSYSDTGELFF
1445	V6-6	J1-4	CASSYSDTGELFF
1434	V6-2	J2-2	CASSYSTTGELFF
1465	V6-4	J2-2	CASSDSGTGELFF
1531	V6-4	J2-2	CASSDSNTGELFF
1449	V6-4	J2-2	CASSDSDTGELFF
1465	V6-4	J2-2	CASSDGDTGELFF
1465	V6-4	J2-2	CASSDRDTGELFF

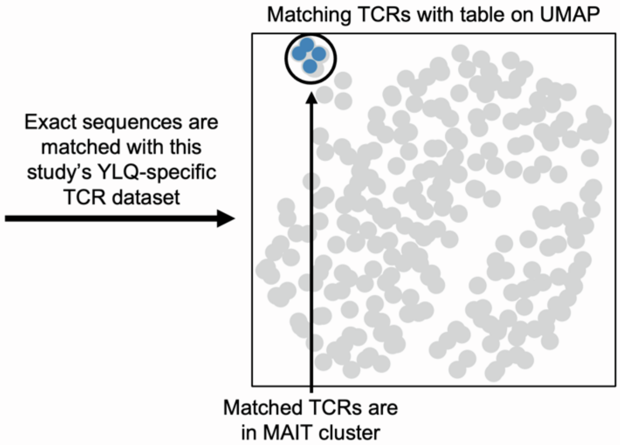

C

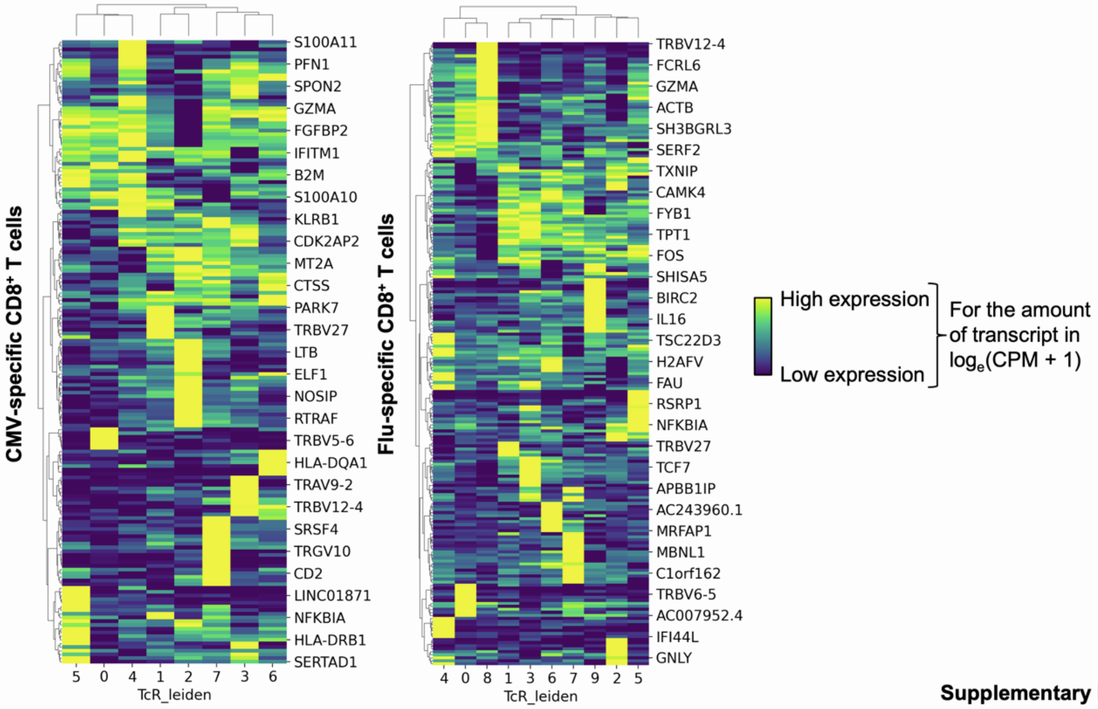

Supplementary Figure 2

Figure S2 (related to Figure 1): Interactions between transcriptomic signatures, TCR clusters, and SARS2-YLQ pMHC-tetramer derived TCRs from literature

(A) Heatmap with rows as YLQ-specific TCR-derived clusters and columns as transcriptomic signatures with values scaled per column, legend on bottom.

(B) Table of *IFNG*-producing TCRs and their respective genes and donors are shown on the left. These sequences are mapped to this study's dataset and matched cells are colored on the right on the TCR-derived UMAP.

(C) Heatmap with rows as individual genes and columns as TCR-derived clusters with values scaled per row. Left heatmap is for CMV-specific cells and right heatmap is for Flu-specific cells, legend on bottom.

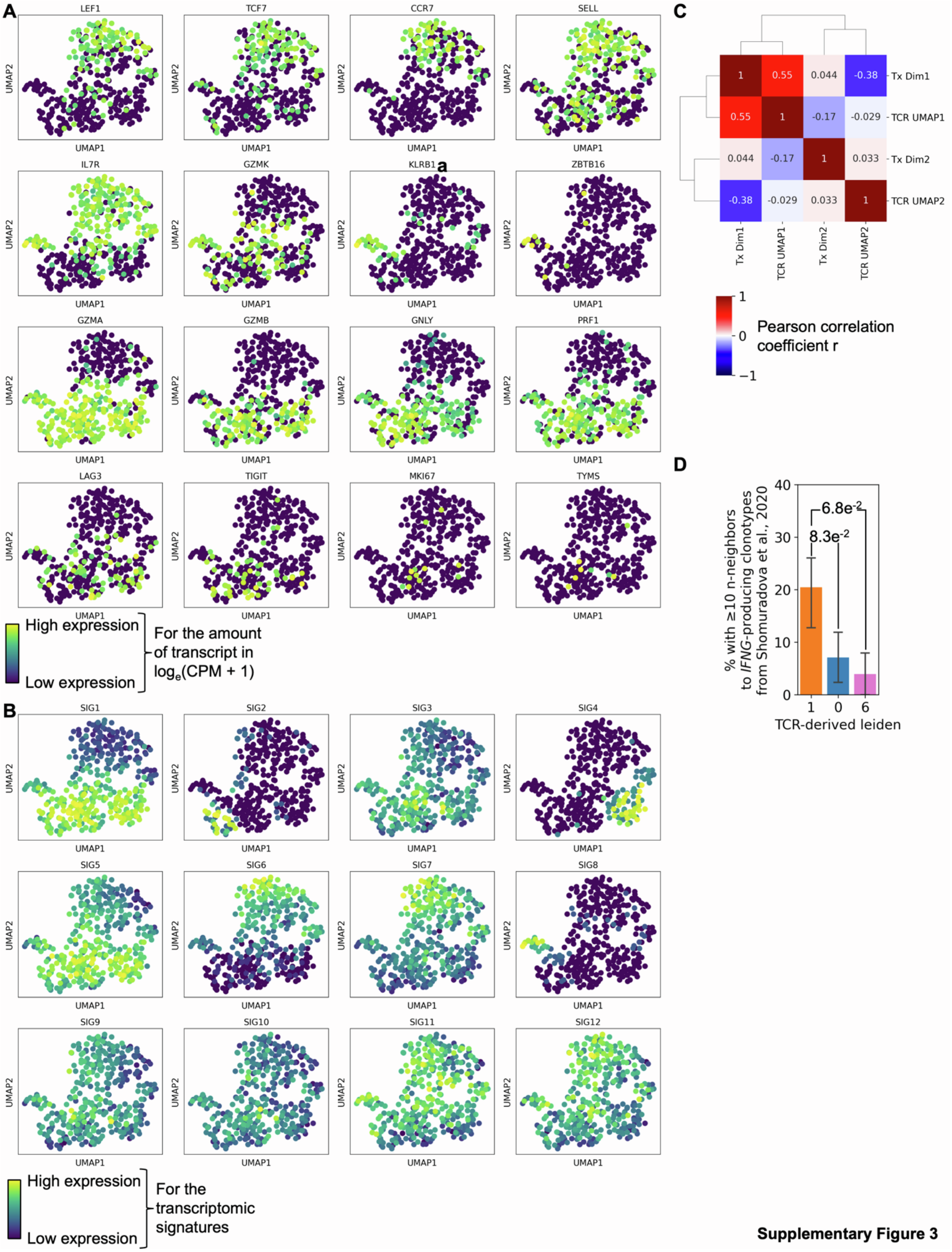

Supplementary Figure 3

Figure S3 (related to Figure 2): Description of RNA UMAP through mRNA, transcriptomic signatures, and comparisons with TCR sequence information

(A) Select mRNA transcripts are plotted on the RNA derived UMAP, legend on bottom.
(B) Transcriptomic signatures are plotted on the RNA derived UMAP, legend on bottom.
(C) Correlation matrix of the positions of TCR clusters in transcriptomic space (RNA UMAP) and in TCR space (TCR UMAPs), legend on right.
(D) Comparison of the TCR sequence similarity of the TCR-derived clusters analyzed in depth in Fig. 2 with literature-derived TCR sequences of *IFNG*-producing YLQ-specific $CD8^+$ T cell clonotypes.

P-values are labeled on respective plots. Error bars on bar plots represent standard error and the bar level represents the mean value of the given variable.

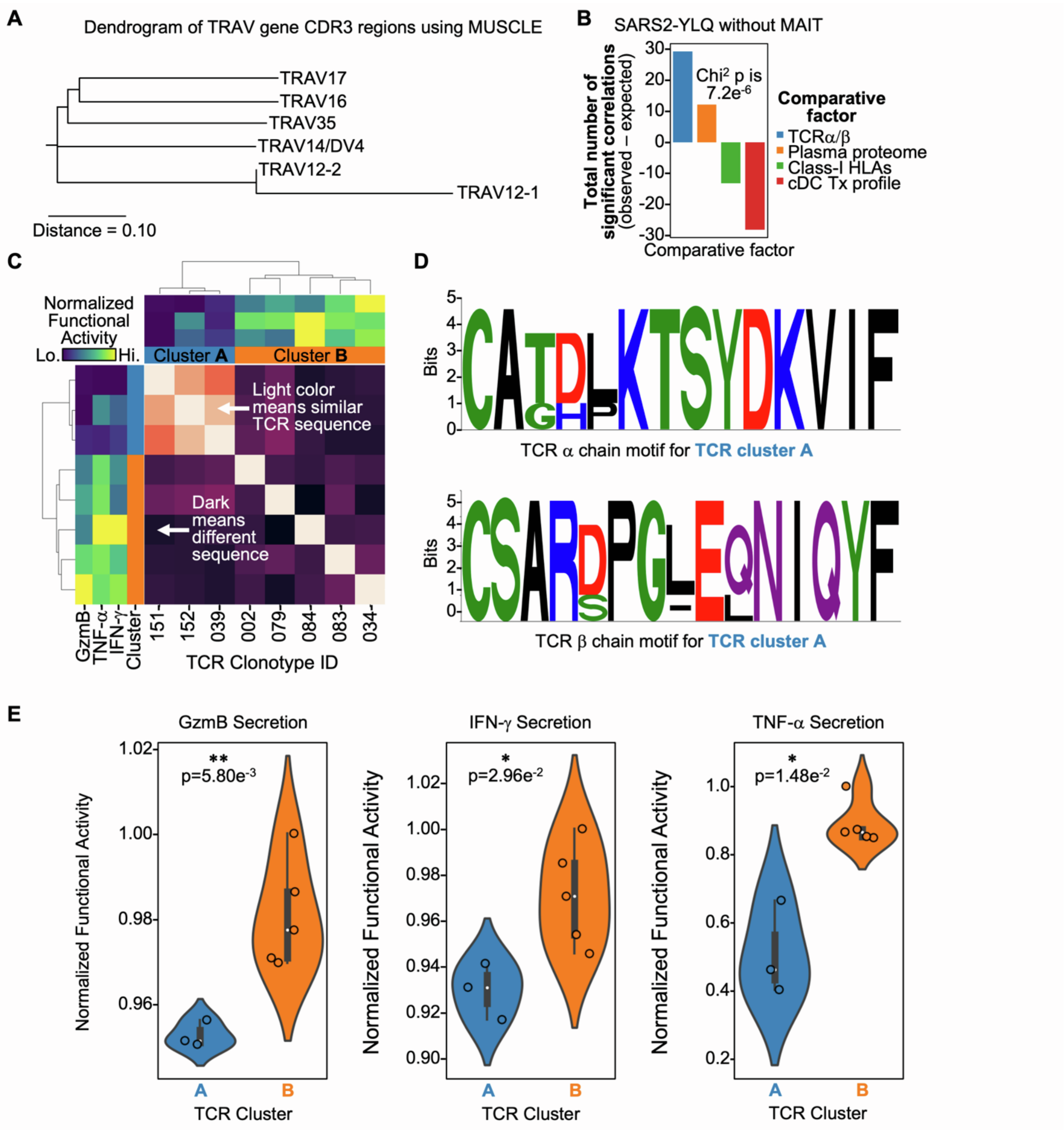

Supplementary Figure 4

Figure S4 (related to Figures 3 and 4): Phylogenetic tree of the CDR3 regions of contraction correlated TRAVs

(A) Dendrogram of a phylogenetic tree computed by MUSCLE describes the relationship between the CDR3 regions of TRAV genes that significantly correlated with the contraction levels of each TCR cluster, legend on bottom.
(B) For SARS2-YLQ specific $CD8^+$ T cells after the removal of MAIT cells from the dataset. Bar plot of each -omic on the x-axis and the total number of observed minus expected significant correlations with unbiased PCA dimensions summed for each -omic on the y-axis; negative bars indicate fewer significant correlations with T cell phenotype than statistically expected while positive bars indicate more significant correlations with phenotype than expected.
(C) Clustered heatmap of pair-wise TCR distances where lighter colors indicate increased TCR sequence similarity and darker colors indicate pairs with dissimilar TCR sequences. Annotated colors above and to the left of the heatmap indicate functional activity with secretion quantified through ELISA, see upper left legend. Cluster colors indicate the two TCR clusters that emerge from this analysis (cluster A in blue and cluster B in orange).
(D) TCR CDR3 α (upper) and β (lower) motifs for TCR cluster A from panel C.
(E) Violin plots with the X-axis as the TCR cluster groups from panel C and the Y-axis as normalized functional activity which is secretion measured via ELISA. Each dot is a clonotype, derived by cloning RLIT-specific HLA-A*02:01 restricted TCRs into primary $CD8^+$ T cells from healthy donors.

P-values are labeled on respective plots with ** for < 0.01 and * for < 0.05. Boxplots denote the medians of the given values and the interquartile range (IQR).

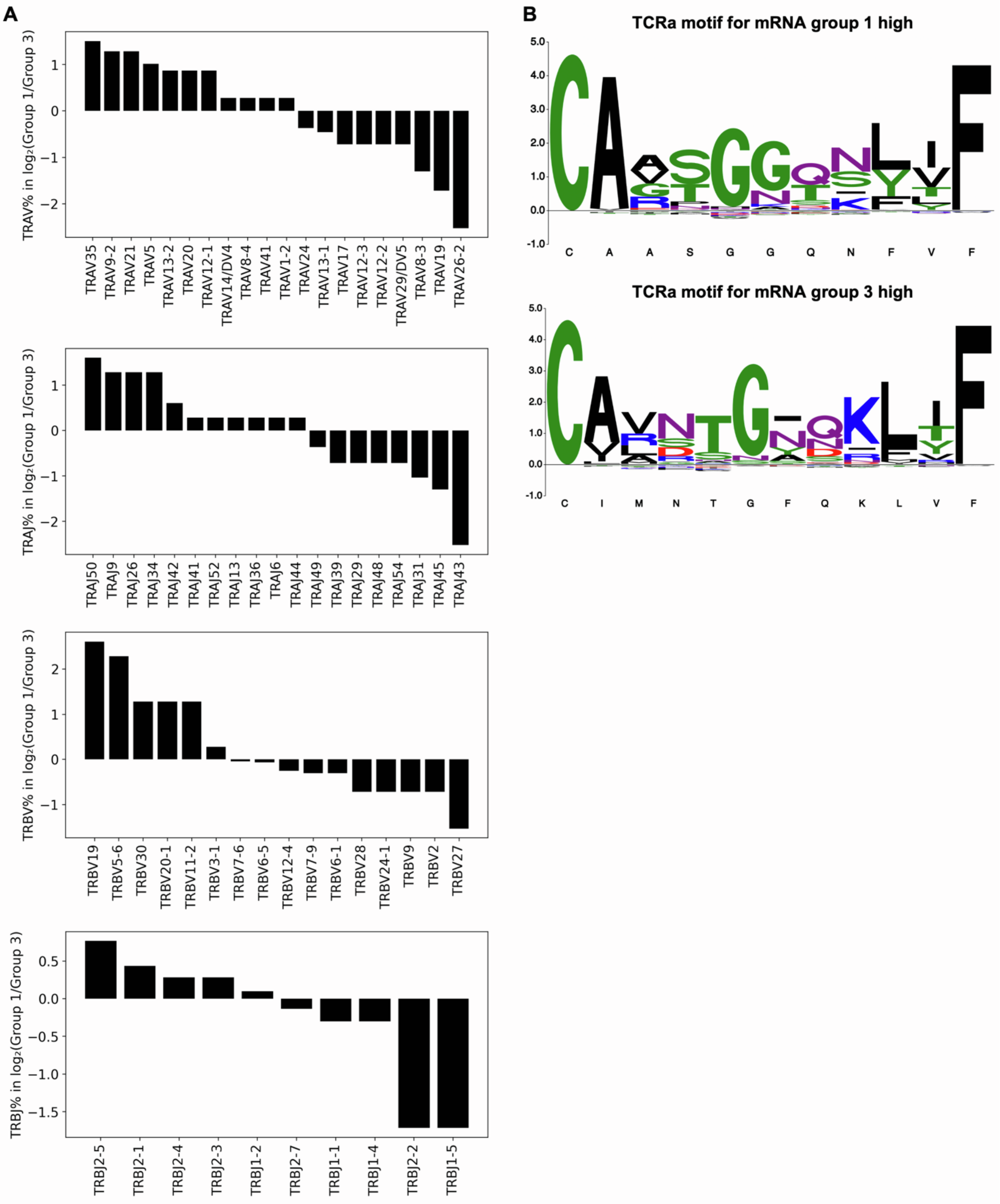

Supplementary Figure 5

Figure S5 (related to Figure 5): TCR sequences separate two distinct bystander activated phenotypes

(A) Bar plot with X-axis as a given TCR gene, the gene is labeled on the y-axis, the values for the y-axis is the log_2(fold change) in the percentage of the given TCR's prevalence in clonotypes from bystander activated cells with high group 1 versus high group 3 mRNA signatures.

(B) TCR α chain sequence motif plots derived from clonotypes from bystander activated cells with high group 1 mRNA signatures on the top and high group 3 mRNA signatures on the bottom.

Supplementary information tables

Table S1 (related to Figure 1): Annotation of pMHC-TCR pair acquisition and retrieval along with TCR clonotype classification and transcriptomic signature description.

Table S2 (related to Figure 2): Description of SARS2-YLQ-specific $CD8^+$ T cell transcriptomic phenotype and correlation with TCR sequence similarity metrics.

Table S3 (related to Figure 3): Description of SARS2-YLQ-specific $CD8^+$ T cell contraction and expansion dynamics and correlation with multi-omic phenotype.

Table S4 (related to Figure 4): Correlation of SARS2-YLQ-specific $CD8^+$ T cell transcriptomic phenotype with their respective TCR sequences and environmental factors.

Table S5 (related to Figure 5): Correlation of CMV-NLV-specific $CD8^+$ T cell transcriptomic and multi-omic phenotype with patient plasma proteomes.
